# Mechanotransduction as a major driver of cell behaviour: mechanisms, and relevance to cell organization and future research

**DOI:** 10.1098/rsob.210256

**Published:** 2021-11-10

**Authors:** Pierre-Henri Puech, Pierre Bongrand

**Affiliations:** Lab Adhesion and Inflammation (LAI), Inserm UMR 1067, CNRS UMR 7333, Aix-Marseille Université UM61, Marseille, France

**Keywords:** signalling, mechanics, cytoskeleton, biomolecular interactions, catch bonds, T lymphocyte activation

## Abstract

How do cells process environmental cues to make decisions? This simple question is still generating much experimental and theoretical work, at the border of physics, chemistry and biology, with strong implications in medicine. The purpose of mechanobiology is to understand how biochemical and physical cues are turned into signals through mechanotransduction. Here, we review recent evidence showing that (i) mechanotransduction plays a major role in triggering signalling cascades following cell–neighbourhood interaction; (ii) the cell capacity to continually generate forces, and biomolecule properties to undergo conformational changes in response to piconewton forces, provide a molecular basis for understanding mechanotransduction; and (iii) mechanotransduction shapes the guidance cues retrieved by living cells and the information flow they generate. This includes the temporal and spatial properties of intracellular signalling cascades. In conclusion, it is suggested that the described concepts may provide guidelines to define experimentally accessible parameters to describe cell structure and dynamics, as a prerequisite to take advantage of recent progress in high-throughput data gathering, computer simulation and artificial intelligence, in order to build a workable, hopefully predictive, account of cell signalling networks.

## 1. Introduction: what is our goal?

Cells continually scan their environment to retrieve guiding cues and take decisions. For example, a cell moving on a solid substrate continually integrates physical and biochemical interactions to control its motion [1]. Also, cells may exert a combination of biochemical interactions and forces to fulfil their function. For example, cytotoxic T cells may use forces to enhance target killing [2] in addition to the local secretion of perforin and delivery of a death signal [3]. Thus, understanding cell function involves the following steps:

— Identify and precisely define the signals generated by cell interaction with the surrounding world. Importantly, these interactions are dependent on both cell structure and dynamics, in particular at the level of the membrane and of the cytoskeleton. They may be dependent on space and time in a complex way. A proper account of this complexity may be required to achieve a detailed description of these signals.
— Identify the sequence and magnitude of intracellular phenomena generated by these signals. During the last two decades, high-throughput methods yielded an enormous amount of data relative to the cell response to a number of stimuli, including formation of molecular complexes and scaffolds (such as so-called signalosomes), structural changes of biomolecules (such as phosphorylation–dephosphorylation events), transmembrane or cytosolic ionic fluxes, and modulation of gene activity leading to differentiation and proliferation events [4–6].
— Build a complete, yet tractable, cell model to relate intracellular phenomena to cellular behaviour changes. While this is currently out of reach, this seems a conceivable long-term prospect in view of recent progress in computer simulation, artificial intelligence and extensive analysis of the response of cell signalling networks to triggering stimuli. However, three difficulties must be overcome beforehand: first, the quality of models is deeply dependent on the pertinence of quantitative parameters used to describe cell structure and function. Second, due to the complexity of cellular systems, available computer power may set a limitation on achievable goals. Finally, experimental data must be accurately fed into the model, which sets a strong need for sensitive and reliable measurements of well-defined quantitative, physical parameters. Such parameters may be from molecular to cellular scale, and their determination requires the combination of a wide range of experimental, numerical and theoretical approaches.

Here, we shall review recent evidence showing that (i) cell behaviour is often strongly influenced by the mechanical properties of their environment; (ii) a number of experiments showed that living cells continually generate and/or feel forces, which strongly influences the conformation of cell components, from the membrane to the proximal cytoskeleton and cytosolic compartments; and (iii) these forces may shape the sensitivity and rapidity of data processing by living cells. It is concluded that the already available wealth of knowledge should be used to select a manageable set of parameters in order to model cell behaviour with the help of recent progresses in quantitative and correlative data analysis including high-throughput data gathering, computer simulation and artificial intelligence.

## 2. Cell behaviour is strongly influenced by mechanical parameters

It is currently well accepted that *cells detect and respond to countless signals* and that *communication between cells in multicellular organisms is mainly mediated by extracellular signal molecules* [7] that interact with receptors mostly located on the cell membrane. It has long been felt that biochemical recognition of soluble mediators such as growth factors, hormones, cell surface-bound molecules such as cadherins, or components of extracellular matrices such as fibronectin or collagen, mediated the generation of the so-called ‘second messengers’ and influenced important events such as cell survival, proliferation, differentiation, shape changes or mediator release. Ligand–receptor binding was thus viewed as the decisive step. However, as acknowledged four decades ago, ‘little was known about cell communication via the direct interaction of molecules bound to the outer surface of cells' ([8], p. 717). During the last two decades, an increasing body of experiments convincingly demonstrated that mechanical^1^ parameters also play an important, sometimes crucial, role in the contact interaction between cells and surface-bound ligands. The following sections give several examples.

### 2.1. Cell survival may require binding to a surface and be influenced by mechanical and geometrical parameters such as substratum stiffness, receptor binding strength and contact area

It has long been recognized that many cell types needed to adhere to solid surfaces to survive and to proliferate. The loss of this property, known as anchorage dependence, was considered a hallmark of transformation into cancer cells [13]. However, while underlying mechanisms were incompletely known, a reasonable view was that adhesion receptors such as integrins, which mediated cell binding to extracellular matrices, displayed conformational changes triggering intracellular signalling cascades upon ligand binding [14]. However, it was rapidly recognized that substratum stiffness strongly influenced the outcome of cell receptor interaction with bound ligands [15,16]. Thus, when renal cells were deposited on collagen-coated hydrogels with an elastic modulus^2^ ranging between 0.2 kPa and 50 kPa, cell proliferation rate increased proportionally to the substrate stiffness [17]. However, tendon-derived stem cells deposited on gelatin hydrogels with a stiffness ranging between about 2 kPa and 25 kPa displayed more active growth on softer surfaces [18]. These results show that cells can, as a whole, sense global mechanical properties of their environment and react to it, and that this reaction may be highly dependent on cell type.

In other experiments, integrin ligands were bound to surfaces with breakable tethers of varying strength: it was found that cell adhesion and spreading required a minimal resistance of about 40 pN per link [19], thus supporting the hypothesis that forces were involved and needed at the cell–substrate interface to trigger cell spreading efficiently.

Finally, it was shown that the survival of adherent cells might require a minimal contact area [20], thus suggesting that cell shape might play a role in linking cell survival to mechanical phenomena [21].

### 2.2. Cell differentiation is dependent on substrate mechanical properties

It has long been shown that differentiation pathways followed by cells growing on surfaces were dependent on the surface structure [22]. However, while this phenomenon was ascribed to the nature of the biomolecules exposed by cell culture substrates, more recent evidence suggested that the substrate stiffness could also deeply influence cell differentiation. Thus, when human mesenchymal stem cells were cultured for several weeks on collagen-coated polyacrylamide gels of varying stiffness [23], cell differentiation pathway was dramatically dependent on substrate mechanical properties, leading to neurons on softer substrates (1 kPa), myoblasts on intermediate substrates (11 kPa) and osteoblasts on stiffer substrates (34 kPa). The importance of matrix stiffness was confirmed by other authors who studied the differentiation of mesenchymal stem cells into smooth muscle or chondrogenic cells in presence of TGF-β [24], osteoblastic or chondrogenic differentiation in the absence of stimuli [25] or adipogenic versus muscle differentiation [26].

In addition to mechanical properties, the geometrical patterning of the surfaces surrounding the cells may also influence their differentiation [27]. Since recent experiments showed that the mesoderm specification of human embryonic stem cells was co-regulated by tensile forces and morphogen gradients [28], it is important to emphasize that contact interactions may generate a complex combination of mechanical and geometrical stimuli, which may make the analysis of mechanical effects more difficult.

### 2.3. Cell migration on a surface may be driven by substrate mechanical properties as well as molecular patterns

Cell migration plays an essential role in development and immunity, as well as in the everyday life of uni- and pluricellular organisms. It has been well established that the migration of motile cells can be guided by chemical cues that may be bound to surfaces [29] or existing as gradients of soluble chemotactic substances [30]; this was felt to involve signals generated by the engagement of cell membrane receptors. Moreover, cells can also react to concentration gradients of adhesive molecules, sometimes in a rather non intuitive way [31]. However, more recently, it was found that substrate mechanical properties were also involved: when fibroblasts were made to migrate on collagen surfaces bound to polyacrylamide hydrogels of heterogeneous stiffness, they were found to accumulate on the stiffer region of the surfaces, a phenomenon that was dubbed durotaxis [32]. While these experiments were performed on separated cells, it was also shown that the speed and persistence of collective cell migration was increased when substrate stiffness was increased [33].

Interestingly, three-dimensional cell migration was well demonstrated to be influenced by environmental topography [34]. Also, it was recently reported that mechanotaxis could direct bacterial motility [35].

### 2.4. A particularly illustrative case: the implication of forces in antigen recognition by T lymphocytes

The detection of foreign antigens by T lymphocytes is a key and early step of the immune response. The cellular and molecular properties of this process are known in considerable detail, which makes it an excellent model to illustrate the mechanisms of cell control [6,36–38]. In addition, this model is well suited to a detailed study of the mechanisms of mechanotransduction, since detection is performed by individual cells, cell responses may be detected very rapidly, and forces play a prominent role in antigen discrimination. The basic principles may be briefly summarized as follows: a typical cell bears on its membrane major histocompatibility complex (MHC)-encoded molecules that expose oligopeptides (p) of about 10 amino acids in length that are randomly derived from the cleavage of intracellular proteins. Thus, only a few copies of a given pMHC complex may be displayed on the whole-cell membrane.^3^ Note that specialized so-called antigen presenting cells (APCs) expose exogenous antigens derived from the intracellular degradation of endocytized material: as such, they act as professional antigen hunters and presenters to the T cells. Since a given T lymphocyte can recognize nearly a single foreign presented peptide, T lymphocytes specific for a given antigen are highly outnumbered by antigen-bearing cells. For an efficient read-out of the presented peptides, an essential requirement is that a T lymphocyte encountering an APC be able to scan nearly all exposed pMHCs within a sufficiently small amount of time. Indeed, as shown with intravital two-photon microscopy, the duration of a typical contact between a T cell and an APC in lymph nodes is of the order of a few minutes [39].

This process was studied *in vitro* by dropping T lymphocytes on artificial surfaces bearing specific ligands of T-cell receptors (TCRs). The detection of TCR ligands results in a sequence of detectable events including rise of intracellular calcium and tyrosine phosphorylations within 4 s [40], modification of membrane dynamics within 10 s [41], extensive cell spreading about 1 min after contact [42,43], production of membrane antigens or soluble mediators within hours, and proliferation within days. This set-up was used to investigate the influence of substrate stiffness on T lymphocyte response. When T lymphocytes were dropped on a substrate coated with a ligand of CD3, a T lymphocyte surface complex tightly associated to the TCR, the efficiency of adhesion and interleukin-2 secretion increased when the substrate elastic modulus was increased from 10 kPa to 200 kPa [44]. In other experiments, T cells were more efficiently induced to release interleukin-2 and proliferate on CD3-binding substrates of 100 kPa that on stiffer substrates of more than 2 MPa elastic modulus [45]. When the range of elasticities and substrate coating were extended, non-monotonic spreading of T cells was observed, suggesting a complex clutch-like molecular behaviour of ligand/receptor/cytoskeleton association [46]. Thus, while cell sensitivity to the stiffness of underlying surfaces supports the involvement of forces in cell function, a quantitative interpretation of experimental data is difficult, since forces generated by cells and membrane molecules exposed to forces are dependent on the receptor ligands exposed by surfaces as well as the mechanical properties of these surfaces. Also, different cell functions may display different force sensitivities [47].

### 2.5. Conclusion

It was well demonstrated during the last two decades that most aspects of cell function, including adhesion, spreading, metabolism, survival or proliferation are influenced by mechanical interactions with their environment. A first step before trying to make sense of available data and integrate them into a general description of cell function may consist of reviewing as quantitatively as possible a selected set of well-studied mechanisms that are likely to play a role in translating mechanical cues into behavioural events.

## 3. How can mechanical phenomena generate or influence signalling cascades?

The basic principle of cell behaviour was first formulated as a two-step process: ‘Initially, an extracellular molecule binds to a specific receptor on a target cell … Subsequently, the receptor stimulates intracellular biochemical pathways leading to a cellular response’ [48]. Presented like this, it may seem a very simple and easily understandable scheme; however, attempts at integrating the details of physical cues into this framework raised many difficulties. Here, we shall review basic cell events involved in the generation of these pathways in order to integrate the contribution and specificity of mechanical events with previously known cell processes.

### 3.1. Cell surface dynamics shape cell–matrix contacts

#### 3.1.1. The surfaces of free cells continually display spontaneous undulations and are decorated by highly dynamic protrusions

Spontaneous movements of the cell membrane have long been observed and quantified in erythrocytes [49] and in many types of nucleated cells [50]. They generate contact and ensuing binding of membrane receptors to bound ligands. These movements may then generate pulling or pushing forces, acting on the ligand–receptor bonds and/or on the membrane. Therefore, a quantitative knowledge of cell surface dynamics is required to understand what is felt by receptors and membranes. We shall describe a few selected examples to provide a feeling for these phenomena. It may be noticed that most information is about isolated cells that are easier to monitor than cells embedded in a large assembly, in particular when shape analysis is necessary.

Several early reports revealed that many nucleated cells displayed continual undulations in the range of tens of nanometres and 1–10 Hz frequency [51–53], and it was likely that these movements might shape the outcome of the interactions between cells and their environment [54]. Moreover, the surface of many cells is dynamically studded with protrusions of diverse shape, such as lamellipodia, blebs, invadopodia, podosomes, filopodia or microvilli, which may preexist to any cell activation (e.g. microvilli on T cells) or be the consequence of an active process (e.g. the lamellipodia on migrating cells). A detailed discussion of the peculiarities of these protrusions would not fall into the scope of this review and we shall focus on filopodia or microvilli. They are long and thin extensions of about 0.1 µm diameter and widely varying length, from 0.5 µm microvilli as found on many lymphocytes [55] to long extensions of up to several tens of micrometres length [56]. Filopodia were defined as exploratory extensions of the cell membrane [57], and they have long been reported to guide neuron growth [56]. Interestingly, they were found to enclose high concentrations of signalling molecules [58] and membrane receptors such as TCRs were found to be concentrated on their tip [59]. Extending and retracting, moving laterally, they provide an ideal platform to probe cell environment, as a blind person would do using a stick while walking or a finger to investigate the surroundings for composition, texture and mechanics.

#### 3.1.2. Cell protrusions such as microvilli may generate contacts of several seconds with presented surfaces

A few selected examples will be described to suggest an order of magnitude for the quantitative properties of short-term cell contacts with their neighbourhood, in terms of space and time.

Interference reflection microscopy (IRM) was used to image the interaction between cells from the human monocytic THP-1 cell line and fibronectin-coated glass surfaces [53]: when cells were dropped on the surface, their underside appeared studded with highly mobile spot-like zones with transverse (vertical) motion of about 10 nm amplitude. Estimated lateral size was of the order of a micrometre but it could not be quantified accurately due to the rather low lateral resolution of IRM. Cells then spread and formed extensive contact areas with surfaces (of the order of 10 µm^2^) during the minute following initial contact, and the contact area displayed oscillations with a typical period of 5–10 s.

Total internal reflection fluorescence microscopy (TIRF) was used to image the interaction between human blood T lymphocytes and surfaces [41]. Cells were labelled with a fluorescent hydrophobic probe to visualize the plasma membrane. When cells were dropped on non-adhesive surfaces, contacts appeared as transient spots of about 0.2 µm^2^ area and an average lifetime of approximately 9 s. If surfaces were coated with a non-activating ligand of T cells, the average duration of these contacts increased to approximately 30 s. If however surfaces were coated with activating antibodies, contacts lasted only approximately 13 s, while robust cell spreading was triggered in the following minutes. These results suggest that T lymphocytes examined surfaces by forming sequential contacts of a few second duration. The same order of magnitude was obtained by a separate group [60]. The analysis suggested that adhesive forces slowed the retraction process and specific stimuli could superimpose an active reaction on this retraction.

Further along these lines, it was attempted to obtain information on the dynamics of membranes in apparent contact with surfaces by quantitative processing of TIRF images [61]. It was concluded that the contacts were mediated by tips of microvilli, which could display fluctuations of about 60 nm amplitude and subsecond period. However, these figures must be considered with caution, since the data processing required some rather strong assumptions, and it must be reminded that membranes are somewhat fuzzy three-dimensional objects, the boundary of which is not clear-cut at the nanometre scale.

#### 3.1.3. Cell protrusions generate forces in the tens of piconewton range

A number of experimental set-ups provided estimates of an order of magnitude for the forces generated on the surface of motile cells. We shall give some examples.

It was reported that a very low hydrodynamic force of the order of 1 pN µm^−1^ could stall the leading edge of fish keratocytes [62]. It was calculated that this was lower than the force required to induce buckling of actin microfilaments, which is consistent with the hypothesis that cells sensed the resisting force and indeed decided to stop.

Neurons were made to contact poorly adhesive microspheres maintained with optical tweezers: as a response, they generated pushing forces of 1–3 pN within 30 ms after contact [63]. Similarly, filopodia of HeLa Cells were brought in contact with adhesive microbeads maintained with optical tweezers: they generated traction forces of the order of 10 pN, depending on the molecules exposed on the microbead surface [64].

Filopodia from a macrophage cell line were reported to retract with a maximum velocity of 600 nm s^−1^ and resist forces of the order of 20 pN; further, the pulling velocity was strongly influenced by applied forces [65]. Thus, the kinetics of cell membrane deformation are also quantitatively influenced by forces applied on cells [66].

When individual T lymphocytes were manoeuvered into contact with activating (CD3/CD28-coated) microbeads, using a BFP [67] to allow force monitoring, they generated pushing (approx. 0.2 pN s^−1^) and pulling (approx. 2 pN s^−1^) forces after a delay of about 1 min [68], consistent with the kinetics of large cytosolic calcium increase often used as an efficient activation reporter [69]. Moreover, human blood T lymphocytes were reported to generate forces of the order of 100 pN on nanopillars of 1 µm diameter after stimulation with CD3 and/or CD28 antibodies [70].

All these examples are consistent with the following picture: small protrusions on the cell membrane continually fluctuate with an amplitude of the order of 10 nm, and generate forces of the order of 10 pN (retraction forces may be substantially higher than pushing forces [71]). The displacement velocity of the tip of these protrusions might be of the order of several hundreds of nm s^−1^. Such light and rapid touches provide an efficient mechanism for spatial scanning of the surface and analysis of its composition and mechanics. However, it is important to emphasize that this simple view is strongly dependent on the time scale of observation. This is very clearly illustrated by an early report on force generation by neurones interacting with microspheres controlled with optical tweezers [72]. As clearly stated by the authors, and evidenced by the power spectrum they provided: ‘the force/velocity relationship might be flat when force and velocity were averaged over 3–5 s, on a finer time scale, random occurrence of fast growth and subsecond retraction became predominent’.

### 3.2. How membrane receptors generate signals: differences between soluble and surface-bound ligands

#### 3.2.1. Cell response to soluble mediators

We shall give a very brief and, as such, incomplete summary of some features that will be contrasted with the outcome of contact-based interactions: cells may indeed use membrane receptors to detect soluble ligands such as hormones. Two main, non-exclusive, mechanisms were shown to trigger intracellular events following receptor–ligand interaction.

*Conformational changes as a primary event*: G protein coupled receptors (GPCRs) are a prominent example as well as one of the largest protein superfamilies [73]. They share a common structure with seven transmembrane domains. Ligand binding to their extracellular part results in a conformational change that will make them interact with intracellular heterotrimeric G proteins (Gαβγ). This event will subsequently activate a number of biochemical pathways [7]. A well-known example is the activation of an adenylate cyclase, resulting in the production of cAMP and protein kinase activation. While these phenomena were initially described as linear sequences of reactions from ligand recognition to second-messenger production, they are now viewed as highly complex signalling networks. It may be noticed that this mechanism is based on an exquisite adaptation of the structure and conformation dynamics of interacting molecules. The difficulty of achieving this matching at the molecular level may explain why this basic mechanism was reused in many circumstances during evolution, thus accounting for the high number of GPCR types.

*Clustering as a primary event*: receptor tyrosine kinases (RTKs) mediate the cell response to many mediators such as growth factors. The cytoplasmic side of these membrane-embedded receptors bears a tyrosine kinase site; ligand binding will trigger a receptor dimerization, allowing a kinase domain to phosphorylate a target domain on the other chain. This triggering mechanism seems less demanding on the ligand–receptor match than conformation-based triggering, and in many cases the simple clustering of receptors with antibodies was found to efficiently activate a signalling pathways. In this regard, T lymphocytes are a representative example: the intracellular part of the TCR–CD3 complex is associated with tyrosine kinases such as p56lck. Receptor clustering will allow this kinase to phosphorylate-specific binding sites called ITAMs (immune receptor tyrosine-associated motives). Phosphorylated ITAMs will then act as docking sites for molecules bearing SH2 domains, rapidly inducing the formation of extensive protein scaffolds (so-called signalosomes) and eventually resulting in T-cell proper activation [37]. The detailed consequences of this fairly general mechanisms will be discussed below. Note that the importance of multimerization is well illustrated by the long known finding that T lymphocytes could be activated by multivalent, but not by monovalent engagement of TCRs by soluble ligands, as revealed by cytosolic calcium increase or medium acidification [74].

#### 3.2.2. Specific mechanisms of cell activation by contact interactions

Both aforementioned mechanisms of cell stimulation may operate during contact interactions of a cell with a solid surface. In addition, the clustering mechanism may be enhanced by the proximity of several receptor–ligands pairs on a surface encountered by a cell. However, it must be emphasized that quantitative differences are expected (and have been experimentally demonstrated) between the so-called three-dimensional interactions (involving at least one freely diffusing molecule) and two-dimensional interactions involving two surface-bound molecular partners [75]. Under two-dimensional conditions, the binding efficiency is dependent on the flexibility of linkage between binding domains and surfaces, as well as movement and deformation, which control the distance between the surfaces, and consequently the duration of ligand–receptor interaction and the forces exerted on receptors.

*Force application on cell membrane components can generate signalling events through conformation changes*. It has been well demonstrated that the application of forces to the cell surface can generate signals. An aspiration force of a few hundreds of piconewtons applied with a micropipette on cells from a leukocyte line was shown to generate a local calcium rise within 10 s, and a subsequent cytoskeletal rearrangement [76]. A stress of 1.8 pN µm^−2^ applied on focal adhesions with magnetic beads triggered Src kinase activation at remote sites of muscle cells within less than one second [77]. Again, T lymphocyte activation provides another striking example. Signalling events could be triggered by subjecting the TCR to mechanical forces. Thus, when optical tweezers were used to apply forces on microbeads bound to T lymphocyte receptors with antibodies binding to different TCR parts, it was found that a force of the order of 50 pN could trigger a rise of intracellular calcium, which is often used as a reporter of T-cell activation. Interestingly, this rise was triggered by a tangential, but not by a normal force, which was indicative of a definite spatial, molecular anisotropy of the phenomenon, consistent with the shearing that may occur during APC scanning by lymphocytes [78]. A similar calcium rise was obtained by applying forces with hydrodynamic flow on T cells bound to surfaces through their TCR [79]. More information was obtained in a later series of experiments [69] using a so-called biomembrane force probe (BFP) [67]. Two micropipettes were used to bring into contact a T lymphocyte and a pMHC-coated microbead bound to a red cell. The red cell, subjected to a controlled aspiration pressure, acted as a tunable, very soft spring allowing to derive the force applied on the bead from the displacement with nearly piconewton sensitivity. The T cell was thus subjected to a series of contact and retraction events which stopped at a prescribed force (a method known as ‘force clamping’), thus applying a constant force to the pMHC-bound TCRs until the bond rupture, and allowing to measure bond lifetime under a given level of force. Intracellular calcium rises were simultaneously recorded with a fluorescent probe. It was concluded that the probability of appearance of calcium rises, viewed as reporters of initial cell activation, was essentially correlated with the total time during periods of 60 s during which the TCR was subjected to a traction force level of 10 pN.

A general mechanism for force-induced signalling may be the force-induced conformation change of specific proteins resulting in the exposure of cryptic docking sites for scaffold and/or signalling proteins. Thus, forces as low as 2 pNs applied on talin, a focal adhesion molecule known to be activated by vinculin, exposed binding sites for vinculin [80]. Interestingly, the structural changes induced by forces on talin may be exquisitely dependent on the kinetics of force application, and talin was suggested to act as a mechanical filter that might discriminate between noise and specific signals [81]. Similarly, structural studies suggested that forces could alter the TCR conformation, resulting in a loosening of the interaction of TCR-associated CD3*ζ* chain and the hydrophobic part of the plasma membrane, thus exposing phophorylable tyrosines to intracellular kinases [82]. Also, the mechanical stretching of detergent-insoluble cytoskeleton structures was shown to induce the binding of a number of proteins such as paxillin or focal adhesion kinase [83]. Thus, there is no doubt that the actomyosin machinery plays an important role in force generation and signalling at the cell–substrate interface. A proteomic study led to the isolation of 905 focal adhesion proteins, 459 of which changed in abundance with myosin II inhibition [84]. Myosin-generated forces could therefore influence the composition of molecular scaffold by influencing the exposure of docking sites, as indicated above, and by acting on molecular interactions [85].

It is important to understand the mechanisms which are potentially involved in the influence of forces on biomolecule interactions; this will be discussed below. A simple thermodynamic argument may support the conclusion that forces of a few pN can significantly alter protein conformation. Integrins are a prominent example of allosteric signalling machines [14]. They may display active and inactive conformations with a major displacement (of the order of 10 nm) of the extracellular domain. A force of 10 pN applied on an integrin may thus generate an energy difference of 100 pN.nm between both conformations, which is 25-fold higher than k_B_T,^4^ the ‘energy unit’ of thermal fluctuations (see above note). Thus, forces generated by cells at interfaces can strongly affect molecular interactions, and hence generate information flow through the formation of molecular assemblies. However, the molecular details of these phenomena have not yet been elucidated despite technical advances, because of their complexity, due to the involvement of hundreds of molecular species at a contact site, a complexity that also increases with the contact duration, since more molecules are gathered or structures formed as the cell actively adapts to stimulation.

Another important mechanism of force-induced signalling involves mechanosensitive membrane-embedded ionic channels [86–88]. Thus, Piezo2 was shown to regulate RhoA and cytoskeletal response through calcium entry [89]*.* A pressure of 100 Pa was sufficient to induce a shift of *Dictyostelium* to bleb-mediated rather than lamellipodial movement [90]. Piezo1 was also implicated in T-cell activation as recently reviewed [91].

*Cell*–*surface contacts may generate signalling cascades by changing the local composition and/or organization of membrane-associated molecules*. It has long been shown that the local composition of cell membranes at interfaces could be different from the bulk membrane composition. This may be explained theoretically [92] and representative examples are the interface between phagocytes and their preys [93], antibody-bearing RBL cells and antigen-bearing vesicles [94], or cytotoxic lymphocytes and their targets [95]. An important mechanism is the steric segregation of molecules with bulky extracellular domains from the close contact regions when surfaces are connected with fairly short adhesion molecules. Thus, CD43 [96] and CD45 [97] are leukocyte membrane molecules with extracellular domains of about 40 nm size, and they were early reported to be excluded from surfaces connected with antibodies [96] or TCR–pMHC links [97]. Interestingly, CD45 bears a phosphatase domain, and CD45 exclusion was found, under certain conditions, to be sufficient to trigger lymphocyte T activation, as a consequence of a displacement of the kinase/phosphatase balance [98,99]. Finally, a close correlation between CD45 exclusion and T-cell activation at interfaces was recently demonstrated experimentally [100]. Two points must be added: first, this intuitively appealing mechanism was suggested theoretically and dubbed as the *kinetic segregation model* [101,102]. It was also shown experimentally that signalling could be impaired when the sorting out of bulky molecules was prevented by replacing native adhesion molecules with engineered molecular linkers of increased length [103,104]. Second, as often occurs in the domain of cell biology, this may not be the whole story since the sorting out of molecules such as CD43 [105] or CD45 [106] may involve active cell phenomena other than mere steric exclusion. Also, it must be emphasized that topographical reorganization of cell membrane molecules such as integrins have long been shown to influence cell function [107].

### 3.3. Local cues drive cell shape, which influences signal processing

While aforementioned data amply demonstrated that contact interactions could strongly influence signalling through mechanical processes, many experiments suggest that this could be achieved in an indirect way through a modulation of membrane curvature and cell shape. Thus, while cell adhesion and spreading are strongly influenced by forces, the ensuing deformations may then strongly influence signalling. Thus, when the adhesiveness of the surfaces was modulated by tuning non-specific physical–chemical properties or the surface density of integrin ligands, it was found that the effect of cell adhesion on cell survival and proliferation was tightly correlated with the induced cell flattening [21,108]. Also, when capillary endothelial cells were deposited on surfaces bearing adhesive patches of varying size and topography, the modulation of cell shape as a consequence of adhesion and spreading was similarly correlated with growth and survival [20]. The shape of a migrating T cell might render it more sensitive to activation cues at its front than at its back, a way to maximize its efficiency in patrolling the body [9,109].

Several theoretical attempts were made in order to provide an explanation for these experimental findings [110–112]. The basic idea was that cell geometry should influence the encounter rate between membrane and cytosolic molecules, with a subsequent alteration of the phosphorylation–dephosphorylation balance, which is thought to play a major role in cell function. Also, cell shape changes and symmetry breaking are often involved in active processes. Thus, the polarization of immune cells towards their targets may play a key role in antigen detection or lethal hit delivery [113].

### 3.4. Conclusion

Mechanical interactions between cells and their environment superimpose the consequences of receptor engagement that have been extensively studied with soluble ligands such as hormones, growth factors or chemotactic substances, and specific mechanisms such as force generation or topographical rearrangement of membrane components.

We shall now review the consequences of these specific mechanisms concerning cell capacity to adapt their responses to an enormous variety of extracellular cues.

## 4. How do intercellular contact interactions shape information retrieval and processing?

Cells detect and respond to countless signals ([7], p. 813). As will be shown in this section, numerous examples suggest that mechanical signals may allow a more efficient spatial and temporal control of extracellular cues, and as a consequence a more efficient discrimination between multiple stimuli at cell/substrate contacts, than soluble mediators.

This may be an essential requirement for cell function due to the following three reasons. First, interactomic studies have well shown that a given protein can interact and react with multiple ligands. As an example, a single integrin such as *α_M_β_2_*, the major receptor primarily expressed on phagocytic leukocytes, was shown to react with many tens of unrelated ligands [114]. As will be discussed below, contact interaction may facilitate ligand discrimination. Second, mechanical stimulation may improve the information flow that is needed by cells to adapt their behaviour [115] and allow more rapid response to stimulation [77]. Third, mechanical interactions might yield more accurate topographical information (e.g. localization) than stimulation of cell membrane receptors with soluble ligands.

We shall now describe selected data demonstrating the importance of force intensity, kinetic accuracy and topographical information that are provided by mechanical signals.

### 4.1. Forces can enhance the cell capacity to discriminate different receptor ligands

TCR-mediated antigen recognition provides a well-studied model of the capacity of forces to enhance ligand discrimination. As mentioned above, T lymphocyte function relies on the cell capacity to recognize within seconds a few or even a single pMHC [116] embedded on a membrane bearing tens of thousands of quite similar molecules [36,38]. While a number of crystallographic studies did not disclose any reproducible conformation change of the TCR that would be due to ligand binding and might explain signalling specificity [117,118], many experimental studies supported the hypothesis [119] that the discrimination efficacy of antigen recognition might be correlated with the physical properties of the TCR–pMHC interaction, more precisely the interaction affinity and lifetime, Indeed, many authors reported a good correlation between the interaction properties of TCR and pMHC as measured with surface plasmon resonance in models including an soluble and a surface-bound reagent and subsequent efficiency of the lymphocyte activation (e.g. interleukin 2 secretion or cell multiplication) [119–122]. More recently, a clever enhancement of TCR–pMHC interaction by addition of a ‘sticky’ peptide was shown to convert a non-stimulatory TCR ligand into a stimulatory one [123]. However, some discrepancies remained unexplained [124], and a theoretical analysis made it clear that it was difficult to understand how recognition sensitivity, specificity and speed could be simultaneously achieved [125]. Indeed, the spontaneous release of a soluble pMHC from a bond made with a TCR is a random event and it may be easily shown that a single lifetime measurement cannot convey sufficiently accurate information on the average lifetime of a given TCR–pMHC interaction [38,126]. However, it was soon recognized that this apparent paradox could be raised by accounting for the occurrence of forces applied *in vivo* on TCR–pMHC bonds. Indeed, the correlation between the bond lifetime and pMHC activation potency was found to be better when experiments were made with surface-bound molecules in presence of forces within the 10 pN range (in flow chambers or BFP experiments) than with soluble molecules in absence of force (e.g. in SPR experiments) [69,127–129]. Also, it was shown theoretically that the application of forces on TCR–pMHC interactions could strongly enhance discrimination by making bond lifetime determination more rapid and more informative [130]. It would clearly be an attractive hypothesis that force-related, possibly small, conformation changes might expose docking sites and trigger biochemical cascades. However, to date this hypothesis has not yet been supported by experimental evidence obtained with X-ray crystallography [117] or cryoelectron microscopy [118], and force-induced structural changes were only related to the binding/unbinding process itself [128,131].

Similarly, B lymphocytes were found to use forces to gather and extract antigens from surfaces [132]. This mechanism was considered to contribute affinity discrimination [132–134] and may constitute a common building block in the mechanisms that leucocytes use to probe the state of the cells they meet and perform, as a consequence, their action.

### 4.2. Mechanical interactions can trigger cell response with ultrashort delay

It is well known that diffusion sets a limitation on the rate of bond formation between soluble molecules [135]. Also, as mentioned earlier, release of a soluble ligand is a random phenomenon, leading statistically to wide variations of the lifetime of individual bonds. However, the encounter of surface-bound ligands and receptors is mainly driven by the motion of surfaces bearing these molecules. As indicated above, there is experimental evidence that cell surfaces continually exhibit nanometre-scale displacements and generate piconewton forces with subsecond period [54,61,72]. Rapidly undulating surfaces might thus generate multiple subsecond contacts in order to achieve a rapid analysis of exposed ligands and behave accordingly. This might involve rapid and transient binding events that were evidenced when ligand–receptor interactions began being studying at the single bond level [136]. Thus, the evidence of ultrashort subsecond binding events between cadherin molecules led to the suggestion that these events might play a role in the subsecond cell adaptation to their environment [137].

In addition, there is now a substantial body of experimental evidence demonstrating the capacity of mechanical interactions to trigger cell response with subsecond delay, here are some examples:

A key step of inflammation is the adhesion of flowing leukocytes to activated endothelial cells in blood capillary vessels. This requires subsecond activation of leukocyte integrins, which was found to be triggered by surface-bound, not soluble chemokines [138].

Magnetic tweezers were used to apply forces on integrin receptors of human airway muscle cells [77]: this resulted in subsecond (less than 0.3 s) Src kinase activation in cell regions distant from the stimulation point, while conventional cell activation with soluble epidermal growth factor did not activate Src even after a 12 s delay. The authors concluded that rapid signal transduction was a unique feature of mechanotransduction.

Muscle cells were subjected to uniaxial loading, through integrin receptors, with a parallel-plate chamber. They were found to adapt force generation to changes of environment stiffness within less than 0.1 s [139].

#### 4.2.1. Conclusion

Following contact interactions, forces can be applied to cell surface receptors with high temporal accuracy and trigger cell responses within seconds. It must be emphasized that this temporal accuracy may be important in view of the growing evidence that the information flow generated by intracellular signals is often finely encoded [140], which means that both the time dependence and amplitude of a signal may convey important pieces of information [141]. This encoding might involve feedback loops of the signalling network and precise characteristics of extracellular stimuli.

### 4.3. Mechanical interactions may be one (and arguably the most efficient) means of providing localization and topographical information

Adequate cell function may require topographical information. Chemotaxis is a prominent example that appeared early in evolution. Bacteria may need to use a concentration gradient to move towards a needed resource. Blood leukocytes may need to move towards foreign or damaged particles that must be ingested.

As will be argued below, while cells may draw topographical information from biochemical cues, mechanical transduction is certainly more efficient to achieve this goal: indeed, bacteria were shown to use repeated determination of chemoattractant concentration to move along a chemical gradient. The basic principle consists of using random directional change (so-called tumbling) at repeated intervals. Receptors are used to measure local chemoattractant concentration at regular intervals. The tumbling frequency increases if the chemoattractant concentration measure decreases. Neutrophils are endowed with a much more elaborate mechanism: they were shown to detect a chemoattractant concentration difference of only 1% between their sides, and then to start moving accordingly [31]. The physical limitations of these processes may be easily understood: the binding of a ligand to a cell receptor is a random event, and many measurements are required for the cell to relate the receptor occupancy to the local ligand concentration, which requires a substantial time to somehow ‘average’ the information or at least allow it to overcome a given level of environmental noise. By contrast, when a membrane receptor of a moving cell encounters a surface-bound ligand, a decision may occur rapidly if not immediately. Thus, a T lymphocyte crawling on a surface may display an immediate stop after encountering its cognate antigen [142].

It may be noticed that the rapidity of cell decision is dependent on the rapidity of intracellular information flow as well as the delay required to locally generate a required piece of information such as the local ligand concentration. In this respect, mechanical transmission of information may be more efficient than the diffusion of signalling molecules such as second messengers or specifically phosphorylated proteins. Interestingly, the two mechanisms are not exclusive. Large scale analysis of interaction maps led to the conclusion that the network of signalling proteins is intimately linked to the cytoskeleton [143], pointing toward nodes of convergence of these two ways of passing the information within the cell.

#### 4.3.1. Conclusion

Contact interactions and mechanotransduction have been shown to convey more information to cells than previously studied mechanisms based on soluble mediators. This information is produced more rapidly and can be transmitted more rapidly within cells. Living cells were shown to take advantage of this performance during information processing. As will be shown below, these features must then be turned into suitable quantitative biophysical parameters in order to improve our understanding of cell function and help us build predictive models of cell behaviour.

## 5. The road ahead

The ultimate goal of cell biologists may well be to achieve a quantitative account of cell function ([7], p. vii) in order to predict the behaviour of cells from their initial state and extracellular signals. In addition, they certainly expect to understand how this cell-scale behaviour is related to the details of cell components at a smaller (e.g. amino acids if not atomic) scale: as stated by Eugene Wigner, a Nobel Prize-winning theoretical physicist, ‘it is nice to know that a computer can understand the problem, but we would like to understand it too’ [144]. How can we proceed?

The outstanding advances of molecular dynamics and the so-called artificial intelligence [145,146] exemplify the power of computer simulation in life sciences. It is now feasible to predict the behaviour of a protein by simulating the dynamics of as much as 100 000 atoms during periods of time sufficiently long to mimic folding and ligand–receptor association.^5^ However, despite previous attempts [147], it does not yet seem feasible to apply this approach to complete, complex cellular systems for three reasons. First, the complexity of these systems is so high that an exhaustive simulation would require a computer power that is not currently available and that might remain insufficient for many decades [148]. Second, even if it was possible to simulate a cellular system, even the most powerful experimental tools currently available would be insufficient to precisely determine and quantify a set of parameters accounting for the initial state [5,6], which may be fluctuating in time even if the cell is in its resting state. Third, the amount of information yielded by such a simulation would require special tools that remain to be established in order to be interpreted [149].

As a consequence, an efficient research strategy suggested by the example of molecular dynamics might consist of achieving a coarse-grained description of cell state and behaviour, starting from a refined set of quantitative parameters sufficiently exhaustive, precisely determined and tractable to take advantage of currently available simulation and measurement tools. We shall now describe two points that should be examined in order to incorporate forces in models of cell function and achieve such a description.

### 5.1. How can we define a minimal set of quantitative parameters to account for ligand–receptor interactions and their modulation by forces?

‘Virtually all proteins function by interacting specifically with other molecules’ [150]. A suitable description of protein–biomolecule interaction is thus an essential requirement to understand cell function. We shall illustrate this statement by describing the progressive refinement of parameters used to describe this phenomenon as a requirement to understand experimental data.

As was stated in *Nature* three decades ago [151], ‘the concept of affinity has long dominated most biological thinking about complex biological reactions even although it was relevant only at equilibrium’. This crucial point is often forgotten. Moreover this conceptual framework was found insufficient when key experiments allowed to elucidate the basic mechanisms of inflammation [152,153]. Blood leukocytes flow in post-capillary venules with a velocity of order of millimetres per second and a wall shear rate of order of hundreds per second.^6^ Local activation of endothelial cells by an inflammatory stimulus makes them bind to flowing leukocytes, resulting in a 100-fold velocity decrease and a jerking motion called *rolling*, followed by a complete arrest and subsequent transmigration across the endothelium. The contact duration between free leukocytes and endothelial cell receptors is less than 0.1 ms before rolling, and less than 10 ms during the rolling phase. The rolling phenomenon was reproduced experimentally in a flow chamber by driving neutrophils along a surface coated with selectin molecules [153]. Definitive arrest was obtained by adding ICAM-1, a ligand of cell integrins, on the chamber floor. The authors concluded that ‘kinetic considerations are essential, since a high on-rate is necessary for efficient interaction of rapidly flowing cells with a substrate, and both rapid on-rate and rapid off-rate are required for rolling’ [153]. This is the reason why it was recognized that the affinity constant could no longer account for all experimental data [151]. This conclusion emphasized the need for experimental determination of the rates of bond formation and rupture between surface-attached molecules. It also soon appeared necessary to assess the influence of forces that were quite high in this experimental situation.

Flow chambers were thus used to study the interaction kinetics of receptor-bearing cells or particles and ligand-coated walls at the single bond level. It was rapidly found that selectins could form short-lived bonds with an off-rate of the order of 1 per second [154,155]. However, this could not be the whole story since it was rapidly reported that even molecules known to induce stable bonds such as antibodies [136], integrins [156] and even the streptavidin–biotin couple [157], a paradigm of a strong binding couple, also generated transient attachments with an off-rate of the order of 1 per second. The two-parameter simplistic framework including an off-rate and an on-rate was therefore found insufficient.

A key parameter that was largely ignored in aforementioned data is the effect of force on bond lifetime. Indeed, it may be estimated that a bond connecting a leukocyte to a wall in presence of a flow with a shear rate of a magnitude similar to that occurring in blood vessels is subjected to a pulling force of several hundreds of piconewtons [136]. A complete description of leukocyte–endothelium interaction in blood should thus account for the effects of forces on bond lifetime. An empirical formula had been early suggested on a theoretical basis by George Bell [158] to relate the off-rate *k*_off_ of a bond subjected to a pulling force *F*:

5.1 $$k_{\mathrm{off}}(F)=k_{\mathrm{off}}(0)\exp\left(\frac{F}{F^{\circ }}\right).$$

Parameter *F°* was interpreted as *k_B_T/a*, where *k_B_* is Boltzmann's constant, *T* the absolute temperature and *a* the distance between the bound state of the ligand and the energy wall resisting detachment. This empirical formula was called Bell's law, and was found to give a satisfactory account of selectin–ligand bonds [159]. Unfortunately, other reports showed that a three-parameter framework, including the bond formation, dissociation rates and Bell's parameter, was, again, insufficient to account for many experimental data for several reasons.

First, as already found on soluble molecules [160], bond formation and rupture between surface-bound receptors and ligands appeared to be a multiphasic phenomenon involving numerous transitions across several, potentially sequential, energy barriers [67,136]. The number of detected barriers might increase in proportion with the resolving power of experimental methods, resulting in a dramatic increase of the number of parameters required to give an accurate description of molecular interactions.^7^ It was suggested to model (i) reaction paths as rugged lines, following a previous suggestion by Zwanzig [161] and (ii) the passage of multiple barriers during bond formation and dissociation as a diffusion process. Recently, the probability *P(t)* that a molecular encounter of (short) duration *t* would lead to bond formation was thus found to vary as *erfc*[(*t_0_*/*t*)^1/2^] [162]. This result could be described qualitatively by concluding that bond formation between two encountering molecules might require a minimal contact time *t_0_*. It gave a satisfactory description of the rate of bond formation between ICAM-1 and an anti-ICAM-1 monoclonal antibody [162,163] and also of the interaction between a TCR and several related pMHCs [138]. The minimal time was found to be of the order of a few milliseconds, which may seem fairly long at molecular scales, but represents a rather intermediate time scale for early recognition at cellular scale.

The concept of a rugged energy landscape as a model for the initial interaction between a receptor and a ligand could also be used to raise what was considered as a paradox, i.e. the finding that the lifetime of a bond appeared dependent on its full history [164,165]. It was suggested to describe this phenomenon as a time dependent bond strengthening that could be added and described by a single parameter, *a*, yielding the following empirical law for the dissociation rate at time *t* of a bond formed at time 0 [166]:

5.2 $$k_{\mathrm{off}}(F,t)=\frac{k_{\mathrm{off}}(F,t=0)}{1+\mathrm{at}}.$$

This formula yields a simple form for bond survival at time *t*, and if gave a satisfactory account of antigen–antibody bonds [166,167].

A second reason is that, while an obvious consequence of Bell's law is that a pulling force should decrease bond lifetime, it was found that the lifetime of some bonds could be increased when they were subjected to a small pulling force [168–170]. These bonds were dubbed catch bonds, following a previous theoretical suggestion from Micah Dembo *et al.* [171]. The initial reports were an incentive to look for a catch-bond behaviour in other ligand–receptor couples, and it was found that a force of the order of 10 pN could substantially increase the lifetime of bonds involving selectins [170], actin [172], cadherins [173], TCR–pMHC [69] or notch receptor [174]. The authors of these reports emphasized the mechanistic importance of this catch-bond phenomenon and concluded that these mechanisms should be considered to account for cell function. Interestingly, it was found that the catch-bond behaviour displayed by an antibody could be described quantitatively with equation (5.2) by assuming a linear force dependence of the bond strengthening parameter [167]. However, the catch-bond nature of TCR/pMHC bond is difficult to fully ascribe to a purely structural feature of the molecules, as suggested by structural studies [128], since it was not always observed when the bond was studied under cell-free conditions [129], and it was reported to depend on intracellular kinase activity [175].

#### 5.1.1. Conclusion

It has now been well demonstrated that the interaction between biomolecules and their ligands at interfaces is a complex and potentially multistep process, the characteristics of which strongly influence cell behaviour. While molecular dynamics can be used to achieve a detailed description of these interactions [131], there is an obvious need for a quantitative description of the behaviour of ligand–receptor couples with a tractable number of reliable and quantitative parameters. This exemplifies the need to describe the molecular properties of cell component with parameters allowing the further connection of atomic-scale phenomena to the behaviour of whole cells. The examples described in this section clearly demonstrated that forces play an important role in influencing molecular interactions that are the basis of cell function.

### 5.2. How can we describe cell states with a proper account of forces?

The concept of cell state is always involved, either implicitly or explicitly, in any analysis of cell function: (a) a cell decision is a transition from an initial to a final state, (b) a signal is supposed to influence cell state, (c) the emergence of any intracellular biochemical cascade is clearly a state change. Therefore, a brief discussion of basic concepts seemed useful in a review of the influence of mechanotransduction on cell function.

Two main methods have been repeatedly used to visualize cell structure and dynamics. Cell state may be viewed as a point in a multidimensional energy-like landscape [176,177]. However, the most frequently used method of summarizing extensive datasets and visualizing cell dynamics probably consists of using graph theory [178] and viewing the signalling machinery as a network with a set of components (nodes) connected (or not) by links called edges which may represent interactions such as binding, activation or inhibition. This procedure was used to represent integrin function [179], protein expression (proteome), phosphorylation [5] and protein interactions (interactome) [6] during T lymphocyte activation. Inspection of the graph may reveal remarkable motives, or subnets that may be hypothesized to fulfil a specific function. A subnet may then be replaced with a node to obtain a simpler representation. This may be called a module [180] that is supposed to function in a relatively independent way, which makes the overall structure more robust, and may allow some reuse of modules (similarly to object oriented computer programming!). This representation can be completed by a definition of the node state (e.g. active or inactive, phosphorylated or not) in order to model the network dynamics [181].

The evidence we summarized here clearly demonstrates that forces may strongly shape the networks currently used to represent cell function, since they are now known to influence molecular interactions that were viewed in particular as reporters of T lymphocyte activation [6]. Forces have been shown to have the capacity to change molecular conformation that may be a basis of the selectivity of phosphorylations [182] that are considered as key components of cell control [5,183]. Finally, forces are known to influence the evolution of cell shape, which in turn has long been shown to influence cell function [108,110].

## 6. General conclusion

Evidence accumulated during the last two decades unambiguously demonstrated that forces play a major role in cell function. It is now known that forces in the order of 1–10 piconewtons with time scales of milliseconds to seconds, and displacements from nanometres to micrometres, are continually generated by cells and their environment. These forces can substantially alter protein conformation and interactions, thus triggering signalling cascades and cell decisions that may be quantitatively modulated by force intensity, kinetics and localization. The current challenge is now to incorporate the wealth of available data into a tractable picture of cell structure and dynamics in order to reach at the same time a fundamental understanding of cell behaviour and a capacity to predict responses to given stimuli.

## Footnotes

1 While this review was essentially focused on the effects of forces on cell behaviour, it was found relevant to mention briefly some specific features of contact interactions, such as the contact location on the cell surface [9], contact area [10] and possibly contact shape [11]. Also, we focused the present review on forces exerted on the cell surface, and, as a direct consequence, the emerging field of *nuclear* mechanotransduction [12] was omitted.

2 The force required to change by a fraction *f* the length of an elastic cylinder of section *s* is the product between *f, s* and the elastic modulus. Thus, the force required to increase or decrease by 10 nm the length of a microvillus-like cylinder of 0.5 µm length, 0.01 µm^2^ section and 1 kPa elastic modulus would be about 0.2 pN resulting in a potential energy change of 1 pN.nm. As a comparison, thermal energy fluctuations are of the order of kBT = 4.1 pN.nm.

3 A cell bearing 100 000 MHC molecules on its surface may thus expose 10 copies of 10 000 pMHC species. Since about 25 000 proteins are encoded by the human genome, a very low number of copies is required to allow the cell to achieve exhaustive exposure of intracellular proteins. It is important that all proteins be represented on each cell in order that a viral infection be detected through the exposure of a least one viral protein. This allows cytotoxic *T* lymphocytes to detect and eliminate virally infected cells, which provides an efficient way to stop viral infections.

4 *k*_B_ is Boltzmann's constant and *T* is the absolute temperature.

5 Note however that some shortcuts such as ‘steered molecular dynamics’ or ‘umbrella sampling’ are still needed to achieve this goal [145,146].

6 The wall shear rate *G* is the velocity gradient: according to basic principles of fluid mechanics, the fluid velocity at a low distance *z* from the blood wall is approximately equal to the product *Gz*.

7 Up to 8 kinetic parameters had been used to account for the interaction between a monoclonal antibody and a small molecule, 2-phenyl-5-oxazolone [160].

## Data accessibility

This article does not contain any additional data.

## References

[1] Valignat M-P, Nègre P, Cadra S, Lellouch AC, Gallet F, Hénon S, Theodoly O. 2014 Lymphocytes can self-steer passively with wind vane uropods. Nat. Com. **5**, 5213. (10.1038/ncomms6213) 25323331

[2] Basu R et al. 2016 Cytotoxic T cells use mechanical force to potentiate target cell killing. Cell **165**, 1-11. (10.1016/j.cell.2016.01.021) PMC4808403 26924577

[3] Kägi D, Vignaux F, Ledermann B, Bürki K, Depraetere V, Nagata S, Hengartner H, Golstein P. 1994 Fas and Perforin pathways as major mechanisms of T cell-mediated cytotoxicity. Science **265**, 528-530. (10.1126/science.7518614) 7518614

[4] Calvano SE et al. 2005 A network-based analysis of systemic inflammation in humans. Nature **437**, 1032-1037. (10.1038/nature03985) 16136080

[5] Tan H et al. 2017 Integrative proteomics and phosphoproteomics profiling reveals dynamic signaling networks and bioenergetics pathways underlying T cell activation. Immunity **46**, 488-503. (10.1016/j.immuni.2017.02.010) 28285833 PMC5466820

[6] Voisinne G et al. 2019 Quantitative interactomics in primary T cells unveils TCR signal diversification extent and dynamics. Nat. Immunol. **20**, 1530-1541. (10.1038/s41590-019-0489-8) 31591574 PMC6859066

[7] Alberts B, Johnson A, Lewis J, Morgan D, Raff M, Roberts K, Walter P. 2015 Molecular biology of the cell, 6th edn. New York, NY: Garland.

[8] Alberts B, Bray D, Lewis J, Raff M, Roberts K, Watson JD. 1983. Molecular biology of the cell, 1st edn. New York, NY: Garland.

[9] Negulescu PA, Krasieva TB, Khan A, Kerschbaum HH, Cahalan MD. 1996 Polarity of T cell shape, motility and sensitivity to antigen. Immunity **4**, 421-430. (10.1016/S1074-7613(00)80409-4) 8630728

[10] Mescher MF. 1992 Surface contact requirements for activation of cytotoxic T lymphocytes. J. Immunol. **149**, 2402-2405. 1527386

[11] Richards DM, Endres RG. 2016 Target shape dependence in a simple model of receptor-mediated endocytosis and phagocytosis. Proc. Natl Acad. Sci. USA **113**, 6113-6118. (10.1073/pnas.1521974113) 27185939 PMC4896703

[12] Athirasala A, Hirsch N, Buxboim A. 2017. Nuclear mechanotransduction: sensing the force from within. Curr. Opin. Cell Biol. **46**, 119-127. (10.1016/j.ceb.2017.04.004) 28641092

[13] Assoian RK. 1997 Anchorage-dependent cell cycle progression. J. Cell. Biol. **136**, 1-4. (10.1083/jcb.136.1.1) 9026502 PMC2132466

[14] Hynes RO. 2002 Integrins: bidirectional allosteric signaling machines. Cell **110**, 673-787. (10.1016/S0092-8674(02)00971-6) 12297042

[15] Geiger B, Spatz JP, Bershadscky AD. 2009 Environmental sensing through focal adhesion. Nat. Rev. Mol. Cell Biol. **10****,** 21-33. (10.1038/nrm2593) 19197329

[16] Hoffman BD, Grashoff C, Schwartz MA. 2011 Dynamic molecular processes mediate cellular mechanotransduction. Nature **475**, 316-323. (10.1038/nature10316) 21776077 PMC6449687

[17] Melica ME, La Regina G, Parri M, Peired AJ, Romagnani P, Lasagni L. 2019. Substrate stiffness modulates renal progenitor cell properties via a ROCK-mediated mechanotransduction mechanism. Cells **8**, 1561. (10.3390/cells8121561) 31816967 PMC6953094

[18] Liu C, Luo J-W, Liang T, Lin L-X, Luo Z-P, Zhuang Y-Q, Sun Y-L. 2018 Matrix stiffness regulates the differentiation of tendon-derived stem cells through FAK-ERK1/2 activation. Exp. Cell Res. **373**, 62-70. (10.1016/j.yexcr.2018.08.023) 30138615

[19] Wang X, Ha T. 2013 Defining single molecular forces required to activate integrin and notch signaling. Science **340**, 991-994. (10.1126/science.1231041) 23704575 PMC3710701

[20] Chen CS, Mrksich M, Huang S, Whitesides GM, Ingber DE. 1997 Geometric control of cell life and death. Science **276**, 1425-1428. (10.1126/science.276.5317.1425) 9162012

[21] Folkman J, Moscona A. 1978 Role of cell shape in growth control. Nature **273****,** 345-349. (10.1038/273345a0) 661946

[22] Kaplan G, Gaudernack G. 1982 In vitro differentiation of human monocytes: differences in monocyte phenotypes induced by cultivation of glass or collagen. J. Exp. Med. **156**, 1101-1114. (10.1084/jem.156.4.1101) 6961188 PMC2186821

[23] Engler AJ, Sen S, Sweeney HL, Discher DE. 2006 Matrix elasticity directs stem cell lineage specification. Cell **126**, 677-689. (10.1016/j.cell.2006.06.044) 16923388

[24] Biomat PJS, Chu JS, Tsou AD, Diop R, Tang Z, Wang A, Li S. 2011 The effect of matrix stiffness on the differentiation of mesenchymal stem cells in response to TGF-β. Biomaterials **32**, 3921-3930. (10.1016/j.biomaterials.2011.02.019) 21397942 PMC3073995

[25] Olivares-Navarrete R, Lee EM, Smith K, Hyzy SL, Doroudi M, Williams JK, Gall K, Boyan BD, Schwartz Z. 2017 Substrate stiffness controls osteoblastic and chondrocytic differentiation of mesenchymal stem cells without exogenous stimuli. PLoS ONE **12**, e0170312. (10.1371/journal.pone.0170312) 28095466 PMC5240960

[26] Xu J et al. 2017 Effect of matrix stiffness on the proliferation and differentiation of umbilical cord mesenchymal stem cells. Differentiation **96**, 30-39. (10.1016/j.diff.2017.07.001) 28753444

[27] Théry M. 2010 Micropatterning as a tool to decipher cell morphogenesis and functions. J. Cell Sci. **123**, 4201-4213. (10.1242/jcs.075150) 21123618

[28] Muncie JM, Ayad NME, Lakins JN, Xue X, Fu J, Weaver VM. 2020 Mechanical tension promotes formation of gastrulation-like nodes and patterns mesoderm specification in human embryonic stem cells. Dev. Cell **55**, 679-694. (10.1016/j.devcel.2020.10.015) 33207224 PMC7755684

[29] Carmeliet P, Tessier-Lavigne M. 2005 Common mechanisms of nerve and blood vessel wiring. Nature **436**, 193-200. (10.1038/nature03875) 16015319

[30] Zigmond SH. 1978 Chemotaxis by polymorphonuclear leukocytes. J. Cell Biol. **77**, 269-287. (10.1083/jcb.77.2.269) 649652 PMC2110037

[31] Luo X, Seveau de Noray V, Aoun L, Biarnes-Pelicot M, Strale P-O, Studer V, Valignat M-P, Theodoly O. 2020 Lymphocytes perform reverse adhesive haptotaxis mediated by LFA-1 integrins. J. Cell Sci. **133**, jcs242883. (10.1242/jcs.242883) 32694167

[32] Lo C-M, Wang H-B, Dembo M, Wang Y-L. 2000 Cell movement is guided by the rigidity of the substrate. Biophys. J. **79****,** 144-152. (10.1016/S0006-3495(00)76279-5) 10866943 PMC1300921

[33] Ng MR, Besser A, Danuser G, Brugge JS. 2012 Substrate stiffness regulates cadherin-dependent collective migration through myosin-II contractility. J. Cell Biol. **199**, 545-563. (10.1083/jcb.201207148) 23091067 PMC3483134

[34] Liu Y-J, Le Berre M, Lautenschlaeger F, Maiuri P, Callan-Jones A, Heuzé M, Takaki T, Voituriez R, Piel M. 2015 Confinement and low adhesion induce fast amoeboid migration of slow mesenchymal cells. Cell **160**, 659-672. (10.1016/j.cell.2015.01.007) 25679760

[35] Kühn M et al. 2021 Mechanotaxis directs *Pseudomonas aeruginosa* twitching motility. Proc. Natl Acad. Sci. USA **118**, e201759118. (10.1073/pnas.2101759118) PMC8325320 34301869

[36] Bongrand P, Malissen B. 1998 Quantitative aspects of T-cell recognition from within the antigen-presenting cell to within the T cell. Bioessays **20**, 412-422. (10.1002/(SICI)1521-1878(199805)20:5<412::AID-BIES8>3.0.CO;2-P) 9670814

[37] Smith-Garvin JE, Koretzky GA, Jordan MS. 2009 T cell activation. Annu. Rev. Immunol. **27**, 591-619. (10.1146/annurev.immunol.021908.132706) 19132916 PMC2740335

[38] Malissen B, Bongrand P. 2015 Early T cell activation: integrating biochemical, structural, and biophysical cues. Annu. Rev. Immunol. **33**, 539-561. (10.1146/annurev-immunol-032414-112158) 25861978

[39] Cahalan MD, Parker I. 2008 Choreography of cell motility and interaction dynamics imaged by two-photon microscopy in lymphoid organs. Annu. Rev. Immunol. **26****,** 85-626. (10.1146/annurev.immunol.24.021605.090620) PMC2732400 18173372

[40] Huse M, Klein LO, Girvin AT, Faraj JM, Li Q-J, Kuhns MS, Davis MM. 2007 Spatial and temporal dynamics of T cell receptor signaling with a photoactivatable agonist. Immunity **27**, 76-88. (10.1016/j.immuni.2007.05.017) 17629516

[41] Brodovitch A, Bongrand P, Pierres A. 2013T lymphocytes sense antigens within seconds and take a decision within one minute. J. Immunol. **191**, 2064-2071. (10.4049/jimmunol.1300523) 23898039

[42] Bunnell SC, Kapoor V, Trible RP, Zhang W, Samelson LE. 2001 Dynamic actin polymerization drives T cell receptor-induced spreading: a role for the signal transduction adaptor LAT. Immunity **14**, 315-329. (10.1016/S1074-7613(01)00112-1) 11290340

[43] Crétel E, Touchard D, Bongrand P, Pierres A. 2011 A new method for rapid detection of T lymphocytes decision to proliferate after encountering activating surfaces. J. Immunol. Methods **364****,** 33-39. (10.1016/j.jim.2010.10.007) 21036178

[44] Judokusumo E, Tabdanov E, Kumari S, Dustin ML, Kam LC. 2012 Mechanosensing in T lymphocyte activation. Biophys. J. **102**, L05-L07. (10.1016/j.bpj.2011.12.011) PMC3260692 22339876

[45] O'Connor RS, Hao X, Shen K, Bashour K, Akimova T, Hancock WW, Kam LC, Milone MC. 2012 Substrate rigidity regulates human T cell activation and proliferation. J. Immunol. **189**, 1330-1339. (10.4049/jimmunol.1102757) 22732590 PMC3401283

[46] Wahl A, Dinet C, Dillard P, Nassereddine A, Puech P-H, Limozin L, Sengupta K. 2019 Biphasic mechanosensitivity of T cell receptor-mediated spreading of lymphocytes. Proc. Natl Acad. Sci. USA **116**, 5908-5913. (10.1073/pnas.1811516116) 30850545 PMC6442626

[47] Saitakis M, Dogniaux S, Goudot C, Bufi N, Asnacios S, Maurin M, Randriamampita C, Asnacios A, Hivroz C. 2017 Different TCR-induced T lymphocyte responses are potentiated by stiffness with variable sensitivity. eLife **6**, e23190. (10.7554/eLife.23190) 28594327 PMC5464771

[48] Pawson T. 1995 Protein modules and signalling networks. Nature **373**, 573-578. (10.1038/373573a0) 7531822

[49] Brochard F, Lennon JF. 1975 Frequency spectrum of the flicker phenomenon in erythrocytes. J. Physique **36**, 1035-1047. (10.1051/jphys:0197500360110103500)

[50] Monzel C, Sengupta K. 2016 Measuring shape fluctuations in biological membranes. J. Phys. D: Appl. Phys. **49**, 243002. (10.1088/0022-3727/49/24/243002)

[51] Krol AY, Grinfeldt MG, Levin SV, Smilgavichus AD. 1990 Local mechanical oscillations of the cell surface within the range 0.2–30 Hz. Eur. Biophys. J. **19**, 93-99. (10.1007/BF00185092) 2073894

[52] Zidovska A, Sackmann E. 2006 Brownian motion of nucleated cell envelopes impedes adhesion. Phys. Rev. Lett. **96**, 048103. (10.1103/PhysRevLett.96.048103) 16486899

[53] Pierres, A, Benoliel AM, Touchard D, Bongrand P. 2008 How cells tiptoe on adhesive surfaces before sticking. Biophys. J. **94**, 4114-4122. (10.1529/biophysj.107.125278) 18234815 PMC2367202

[54] Pierres A, Monnet-Corti V, Benoliel AM, Bongrand P. 2009 Do membrane undulations help cells probe the world ? Trends Cell Biol. **19**, 428-433. (10.1016/j.tcb.2009.05.009) 19709883

[55] Majstoravich S, Zhang J, Nicholson-Dykstra S, Linder S, Friedrich W, Siminovitch KA, Higgs HN. 2004 Lymphocyte microvilli are dynamic, actin-dependent structures that do not require Wiskott-Aldrich syndrome protein (WASp) for their morphology. Blood **104**, 1396-1403. (10.1182/blood-2004-02-0437) 15130947

[56] Bentley D, Toroian-Raymond A. 1986 Disoriented pathfinding by pioneer neurone growth cones deprived of filopodia by cytochalasin treatment. Nature **323**, 712-715. (10.1038/323712a0) 3773996

[57] Ridley AJ. 2011 Life at the leading edge. Cell **145**, 1012-1022. (10.1016/j.cell.2011.06.010) 21703446

[58] Johnson HE, Haugh JM. 2016 Are filopodia privileged signaling structures in migrating cells? Biophys. J. **111****,** 1827-1830. (10.1016/j.bpj.2016.09.022) 27712827 PMC5103009

[59] Jung Y, Riven I, Feigelson SW, Kartvelishvily E, Tohya K, Miyasaka M, Alon R, Haran G. 2016 Three-dimensional localization of T-cell receptors in relation to microvilli using a combination of supperresolution microscopies. Proc. Natl Acad. Sci. USA **113**, E5916-E5924. (10.1073/pnas.1605399113) 27647916 PMC5056101

[60] Cai E et al. 2017 Visualizing dynamic microvillar search and stabilization during ligand detection by T cells. Science **356**, aa3118. (10.1126/science.aal3118) PMC6364556 28495700

[61] Brodovitch A, Limozin L, Bongrand P, Pierres A. 2015 Use of TIRF to monitor T-lymphocyte membrane dynamics with submicrometer and subsecond resolution. Cell. Mol. Bioeng. **8**, 178-186. (10.1007/s12195-014-0361-8) 25798205 PMC4361759

[62] Bohnet S, Ananthakrishnan R, Mogilner A, Meister J-J, Verkhovsky AB. 2006 Weak forces stalls protrusion at the leading edge of the lamellipodium. Biophys. J. **90**, 1810-1820. (10.1529/biophysj.105.064600) 16326894 PMC1367330

[63] Cojoc D, Difato F, Ferrari E, Shahapure RB, Laishram J, Righi M, di Fabrizio EM, Torre V. 2012 Properties of the force exerted by filopodia and lamellipodia and the involvement of cytoskeletal components. PLoS ONE **2**, e1072. (10.1371/journal.pone.0001072) PMC2034605 17957254

[64] Romero S, Quatela A, Bornschlög T, Guadagnini S, Bassereau P, Tran Van Nhieu G. 2012 Filopodium retraction is controlled by adhesion to its tip. J. Cell Sci. **125**, 4999-5004. (10.1242/jcs.104778) 22899718 PMC3533388

[65] Kress H, Stelzer EHK, Holzer D, Buss F, Griffiths G, Rohrbach A. 2007 Filopodia act as phagocytic tentacles and pull with discrete steps and a load-dependent velocity. Proc. Natl Acad. Sci. USA **104**, 11 633-11 638. (10.1073/pnas.0702449104) PMC1913848 17620618

[66] Zucchetti AE, Paillon N, Markova O, Dogniaux S, Hivroz C, Husson J. 2021 Influence of external forces on actin-dependent T cell protrusions during immune synapse formation. Biol. Cell **113**, 250-263. (10.1111/boc.202000133) 33471387

[67] Merkel R, Nassoy P, Leung A, Ritchie K, Evans E. 1999 Energy landscapes of receptor-ligand bonds explored with dynamic force spectroscopy. Nature **397**, 50-53. (10.1038/16219) 9892352

[68] Husson J, Chemin K, Bohineust A, Hivroz C, Henry N 2011 Force generation upon T cell receptor engagement. PLoS ONE **6**, e19680. (10.1371/journal.pone.0019680) 21572959 PMC3091878

[69] Liu B, Chen W, Evavold BD, Zhu C. 2014 Accumulation of dynamic catch bonds between TCR and agonist peptide-MHC triggers T cell signaling. Cell **157**, 357-368. (10.1016/j.cell.2014.02.053) 24725404 PMC4123688

[70] Bashour KT, Gondarenko A, Chen H, Shen K, Liu X, Huse M, Hone JC, Kam LC. 2014 CD28 and CD3 have complementary roles in T-cell traction forces. Proc. Natl Acad. Sci. USA **111**, 2241-2246. (10.1073/pnas.1315606111) 24469820 PMC3926067

[71] Sawicka A, Babataheri A, Dogniaux S, Barakat AI, Gonzalez-Rodriguez D, Hivroz C, Husson J. 2017 Micropipette force probe to quantify single-cell force generation: application to T-cell activation. Mol. Biol. Cell **28**, 3229-3239. (10.1091/mbc.E17-06-0385) 28931600 PMC5687025

[72] Shahapure R, Difato F, Laio A, Bisson G, Ercolini E, Amin L, Ferrari E, Torre V. 2010 Force generation in lamellipodia is a probabilistic process with fast growth and retraction events. Biophys. J. **98**, 979-988. (10.1016/j.bpj.2009.11.041) 20303855 PMC2849058

[73] Inoue A et al. 2019 Illuminating G-protein-coupling selectivity of GPCRs. Cell **177**, 1933-1947. (10.1016/j.cell.2019.04.044) 31160049 PMC6773469

[74] Boniface JJ et al. 1998 Initiation of signal transduction through the T cell receptor requires the multivalent engagement of peptide/MHC ligands. Immunity **9**, 459-466. (10.1016/S1074-7613(00)80629-9) 9806632

[75] Pierres A, Benoliel AM, Zhu C, Bongrand P. 2001 Diffusion of microspheres in shear flow near a wall use to measure binding rates between attached molecules. Biophys. J. **81**, 25-42. (10.1016/S0006-3495(01)75677-9) 11423392 PMC1301489

[76] Horoyan M, Benoliel A-M, Capo C, Bongrand P. 1990 Localization of calcium and microfilament changes in mechanically stressed cells. Cell Biophys. **17**, 243-256. (10.1007/BF02990720) 1714350 PMC2723714

[77] Na S, Collin O, Chowdhury F, Tay B, Ouyang M, Wang Y, Wang N. 2008 Rapid signal transduction in living cell is a unique feature of mechanotransduction. Proc. Natl Acad. Sci. USA **105**, 6626-6631. (10.1073/pnas.0711704105) 18456839 PMC2373315

[78] Kim ST, Takeuchi K, Sun Z-YJ, Touma M, Castro CE, Fahmy A, Lang MJ, Wagner G, Rienherz EL. 2009 The ab T cell receptor is an anisotropic mechanosensor. J. Biol. Chem. **284**, 31 028-31 037. (10.1074/jbc.M109.052712) PMC2781503 19755427

[79] Li Y-C, Chen B-M, Wu P-C, Cheng T-L, Kao L-S, Tao M-H, Lieber A, Roffler SR. 2010 Cutting edge: mechanical forces acting on T cells immobilized via the TCR complex can trigger TCR signaling. J. Immunol. **184**, 5959-5963. (10.4049/jimmunol.0900775) 20435924

[80] Del Rio A, Perez-Jimenez R, Liu R, Roca-Cusachs P, Fernandez JM, Sheetz MP. 2009 Stretching single talin rod molecules activates vinculin binding. Science **323**, 638-641. (10.1126/science.1162912) 19179532 PMC9339221

[81] Tapia-Rojo R, Alonso-Caballero A, Fernandez JM. 2020 Taling folding as the tuning fork of cellular mechanotransduction. Proc. Natl Acad. Sci. USA **117**, 21 346-21 353. (10.1073/pnas.2004091117) 32817549 PMC7474635

[82] Brazin KN et al. 2018 The T cell antigen receptor a transmembrane domaine coordinates triggering regulation of bilayer immersion and CD3 subunit associations. Immunity **49**, 829-841. (10.1016/j.immuni.2018.09.007) 30389415 PMC6249037

[83] Sawada Y, Sheetz MP. 2002 Force transduction by triton cytoskeletons. J. Cell Biol. **156**, 609-615. (10.1083/jcb.200110068) 11839769 PMC2174068

[84] Kuo J-C, Han X, Hsiao C-T, Yates III JR, Waterman CM. 2011 Analysis of the muosin-II-responsive focal adhesion proteome reveals a role for b-pix in negative regulation of focal adhesion maturation. Nat. Cell Biol. **13**, 383-393. (10.1038/ncb2216) 21423176 PMC3279191

[85] Wolfenson H, Bershadsky A, Henis YI, Geiger B. 2011 Actomyosin-generated tension controls the molecular kinetics of focal adhesion. J. Cell Sci. **124**, 1425-1432. (10.1242/jcs.077388) 21486952 PMC3078811

[86] Chalfie M. 2009 Neurosensory mechanotransduction. *Nature Rev*. Mol. Cell Biol. **10**, 44-52. (10.1038/nrm2595) 19197331

[87] Berrier C, Pozza A, de Lacroix de Lavalette A, Chardonnet S, Mesneau A, Jaxel C, le Maire M, Ghazi A. 2013 The purified mechanosensitive channel TREK-1 is directly sensitive to membrane tension. J. Biol. Chem. **288**, 27 307-27 314. (10.1074/jbc.M113.478321) PMC3779726 23897808

[88] Douguet D, Honoré E. 2019 Mammalian mechanoelectrical transduction: structure and function of force-gated ion channels. Cell **179**, 340-354. (10.1016/j.cell.2019.08.049) 31585078

[89] Pardo-Pastor C et al. 2018 Piezo2 channel regulates RhoA and actin cytoskeleton to promote cell mechanobiological responses. Proc. Natl Acad. Sci. USA **115**, 1925-1930. (10.1073/pnas.1718177115) 29432180 PMC5828612

[90] Srivasta N, Traynor D, Piel M, Kabla AJ, Kay RR. 2020 Pressure sensing through piezo channels controls whether cells migrate with blebs or pseudopods. Proc. Natl Acad. Sci. USA **117**, 2506-2512. (10.1073/pnas.1905730117) 31964823 PMC7007555

[91] Liu CSC, Ganguly D. 2019 Mechanical cues for T cell activation: role of piezo1 mechanosensors. Crit. Rev. Immunol. **39****,** 15-38. (10.1615/CritRevImmunol.2019029595) 31679192

[92] Bell GI, Dembo M, Bongrand P. 1984 Competition between nonspecific repulsion and specific bonding. Biophys. J. **45**, 1051-1064. (10.1016/S0006-3495(84)84252-6) 6743742 PMC1434996

[93] Tsan M-F, Berlin RD. 1971 Effect of phagocytosis on membrane transport of nonelectrolytes. J. Exp. Med. **134**, 1016-1025. (10.1084/jem.134.4.1016) 5098055 PMC2138989

[94] McCloskey MA, Poo MM. 1986 Contact-induced redistribution of specific membrane components: local accumulation and development of adhesion. J. Cell Biol. **102**, 2185-2196. (10.1083/jcb.102.6.2185) 2423534 PMC2114238

[95] Andre P, Benoliel A-M, Capo C, Foa C, Buferne M, Boyer C, Schmitt-Verhulst A-M, Bongrand P. 1990 Use of conjugates made between a cytolytic T cell clone and target cells to study the redistribution of membrane molecules in contact areas. J. Cell Sci. **97**, 335-347. (10.1242/jcs.97.2.335) 2277095

[96] Soler M, Merant C, Servant C, Fraterno M, Allasia C, Lissitzky JC, Bongrand P, Foa C. 1997 Leukosialin (CD43) behavior during adhesion of human monocytic THP-1 cells to red blood cells. J. Leukocyte Biol. **61**, 609-618. (10.1002/jlb.61.5.609) 9129210

[97] Leupin O, Zaru R, Laroche T, Müller S, Valitutti S. 2000 Exclusion of CD45 from the T-cell receptor signaling area in antigen-stimulated T lymphocytes. Curr. Biol. **10****,** 277-290. (10.1016/S0960-9822(00)00362-6) 10712909

[98] James JR, Vale RD. 2012 Biophysical mechanism of T-cell receptor triggering in a reconstituted system. Nature **487****,** 64-69. (10.1038/nature11220) 22763440 PMC3393772

[99] Chang VT et al. 2019 Initiation of T cell signaling by CD45 segregation at 'close contacts'. Nat. Immunol. **17****,** 574-582. (10.1038/ni.3392) PMC4839504 26998761

[100] Razvag Y, Neve-Oz Y, Sajman J, Yakovian O, Reches M, Sherman E. 2019 T-cell activation through isolated tight contacts. Cell Rep. **29**, 3506-3521. (10.1016/j.celrep.2019.11.022) 31825832

[101] Davis SJ, van der Merwe PA. 2006 The kinetic-segregation model: TCR triggering and beyond. Nat. Immunol. **7**, 803-809. (10.1038/ni1369) 16855606

[102] Burroughs NJ, Lazic Z, van der Merwe PA. 2006 Ligand detection and discrimination by spatial relocalization: a kinase-phosphatase segregation model of TCR activation. Biophys. J. **14**, 315-329. (10.1529/biophysj.105.080044) PMC1544308 16751250

[103] Milstein O et al. 2008 Nanoscale increases in CD2-CD48-mediated intermembrane spacing decrease adhesion and reorganize the immunological synapse. J. Biol. Chem. **283**, 34 414-34 422. (10.1074/jbc.M804756200) PMC2590684 18826951

[104] Bakalar MH, Joffe AM, Schmid EM, Son S, Podolski M, Fletcher DA. 2018 Size-dependent segregation controls macrophage phagocytosis of antibody-opsonized targets. Cell **174**, 131-142. (10.1016/j.cell.2018.05.059) 29958103 PMC6067926

[105] Delon J, Kaibuchi K, Germain RN. 2001 Exclusion of CD43 from the immunological synapse is mediated by phosphorylation-regulated relocation of the cytoskeletal adaptor moesin. Immunity **15**, 691-701. (10.1016/S1074-7613(01)00231-X) 11728332

[106] Jung Y, Wen L, Altman A, Ley K. 2021 CD45 pre-exclusion from the tips of T cell microvilli prior to antigen recognition. Nat. Com. **12**, 3872. (10.1038/s41467-021-23792-8) PMC8222282 34162836

[107] Maheshwari G, Brown G, Lauffenburger DA, Wells A, Griffith LG. 2000 Cell adhesion and motility depend on nanoscale RGD clustering. J. Cell Sci. **113**, 1677-1686. (10.1242/jcs.113.10.1677) 10769199

[108] Ingber DE et al. 1990 Fibronectin controls capillary endothelial cell growth by modulating cell shape. Proc. Natl Acad. Sci. USA **87**, 3579-3583. (10.1073/pnas.87.9.3579) 2333303 PMC53945

[109] Wei X, Tromberg BJ, Cahalan MD. 1999 Mapping the sensitivity of T cells with a optical trap: polarity and minimal number of receptors for Ca^2+^ signaling. Proc. Natl Acad. Sci. USA **96**, 8471-8476. (10.1073/pnas.96.15.8471) 10411899 PMC17540

[110] Meyers J, Craig J, Odde DJ. 2006 Potential for control of signaling pathways via cell size and shape. Curr. Biol. **16**, 1685-1693. (10.1016/j.cub.2006.07.056) 16950104

[111] Haugh JM. 2007 Membrane-binding/modification model of signaling protein activation and analysis of its control by cell morphology. Biophys. J. **92**, PL93-L95. (10.1529/biophysj.107.105213) PMC1868972 17416624

[112] Neves SR et al. 2008 Cell shape and negative links in regulatory motifs together control spatial information flow in signaling networks. Cell **133**, 666-680. (10.1016/j.cell.2008.04.025) 18485874 PMC2728678

[113] Sims TN et al. 2007 Opposing effects of PKCt and Wasp on symmetry breaking and relocation of the immunological synapse. Cell **129**, 773-785. (10.1016/j.cell.2007.03.037) 17512410

[114] Yakubenko VP, Lishko VK, Lam SCT, Ugarova TP. 2002 A molecular basis for integrin α_M_β_2_ ligand binding promiscuity. J. Biol. Chem. **277**, 483 635-448 642. (10.1074/jbc.M208877200) 12377763

[115] Deneke VE, Di Talia S. 2018 Chemical waves in cell and developmental biology. J. Cell Biol. **217**, 1193-1204. (10.1083/jcb.201701158) 29317529 PMC5881492

[116] Sykulev Y, Joo M, Vturina I, Tsomides TJ, Eisen HN. 1996 Evidence that a single peptide-mhc complex on a target cell can elicit a cytolytic T cell response. Immunity **4**, 565-571. (10.1016/S1074-7613(00)80483-5) 8673703

[117] Rudolph MG, Stanfield RL, Wilson IA. 2006 How TCRs bind MHCs, peptides, and coreceptors. Annu. Rev. Immunol. **24**, 419-466. (10.1146/annurev.immunol.23.021704.115658) 16551255

[118] Mariuzza RA, Agnihotri P, Orban J. 2020 The structural basis of T-cell receptor (TCR) activation: an enduring enigma. J. Biol. Chem. **295**, 914-925. (10.1074/jbc.REV119.009411) 31848223 PMC6983839

[119] Matsui K, Boniface JJ, Steffner P, Reay PA, Davis MM. 1994 Kinetics of T-cell receptor binding to peptide/I-E k complexes: correlation of the dissociation rate with T-cell responsiveness. Proc. Natl Acad. Sci. USA **91**, 12 862-12 866. (10.1073/pnas.91.26.12862) PMC45540 7809136

[120] Alam SM, Travers PJ, Wung JL, Nasholds W, Redpath S, Jameson SC, Gascoigne NRJ. 1996 T-cell-receptor affinity and thymocytes positive selection. Nature **381**, 616-620. (10.1038/381616a0) 8637599

[121] Lyons DS, Lieberman SA, Hampl J, Boniface JJ, Chien Y-H, Berg LJ, Davis MM. 1996 A TCR binds to antagonist ligands with lower affinities and faster dissociation rates than to agonists. Immunity **5**, 53-61. (10.1016/S1074-7613(00)80309-X) 8758894

[122] Aleksic M, Dushek O, Zhang H, Shenderov E, Chen J-L, Cerundolo V, Coombs V, Coombs D, van der Merwe PA. 2010 Dependence of T cell antigen recognition on T cell receptor-peptide MHC confinement time. Immunity **32**, 163-174. (10.1016/j.immuni.2009.11.013) 20137987 PMC2862301

[123] Gee MH, Sibener LV, Birnbaum ME, Jude KM, Yang X, Fernandes RA, Mendoza JL, Glassman CR, Garcia KC. 2018 Stress-testing the relationship between T cell receptor/peptide-MHC affinity and cross-reactivity using peptide velcro. Proc. Natl Acad. Sci. USA **115**, E7369-E7378. (10.1073/pnas.1802746115) 30021852 PMC6077720

[124] Rosette C, Werlen G, Daniels MA, Holman PO, Alam SM, Travers PJ, Gascoigne NRJ, Palmer E, Jameson SC. 2001 The impact of duration versus extent of TCR occupancy on T cell activation: a revision of the kinetic proofreading model. Immunity **15**, 59-70. (10.1016/S1074-7613(01)00173-X) 11485738

[125] Mckeithan TW. 2005 Kinetic proofreading in T-cell receptor signal transduction. Proc. Natl Acad. Sci. USA **92**, 5042-5046. (10.1073/pnas.92.11.5042) PMC41844 7761445

[126] He H-H, Bongrand P. 2012 Membrane dynamics shape TCR-generated signaling. Front. Immunol. **3**, 90. (10.3389/fimmu.2012.00090) 22566969 PMC3342369

[127] Robert P, Aleksic M, Dushek O, Cerundolo V, Bongrand P, van der Merwe A. 2012 Kinetics and mechanics of 2D interactions between T cell receptors and different activating ligands. Biophys. J. **102**, 248-257. (10.1016/j.bpj.2011.11.4018) 22339861 PMC3260781

[128] Sibener LV et al. 2018 Isolation of a structural mechanism for uncoupling T cell receptor signaling from peptide-MHC binding. Cell **174**, 672-687. (10.1016/j.cell.2018.06.017) 30053426 PMC6140336

[129] Limozin L, Bridge M, Bongrand P, Dushek O, van der Merwe PA, Robert P. 2019 TCR-pMHC kinetics under force in a cell-free system show no intrinsic catch bond, but a minimal encounter duration before binding. Proc. Natl Acad. Sci. USA **116**, 16 943-16 948. (10.1073/pnas.1902141116) PMC6708305 31315981

[130] Klotzsch E, Schütz GJ. 2013 Improved ligand discrimination by force-induced unbinding of the T cell receptor from peptide-MHC. Biophys. J. **104**, 1670-1675. (10.1016/j.bpj.2013.03.023) 23601314 PMC3628311

[131] Hwang W, Mallis RJ, Lang MJ, Reinherz EL. 2020 The *αβ*TCR mechanosensor exploits dynamic ectodomain allostery to optimize its ligand recognition site. Proc. Natl Acad. Sci. USA **117**, 21 336-21 345. (10.1073/pnas.2005899117) 32796106 PMC7474670

[132] Kumari A et al. 2019 Actomyosin-driven force patterning controls endocytosis at the immune synapse. Nat. Com. **10**, 2870. (10.1038/s41467-019-10751-7) PMC6599028 31253773

[133] Natkanski E, Lee W-Y, Mistry B, Casal A, Molloy JE, Tolar P. 2013 B cells use mechanical energy to discriminate antigen affinities. Science **340**, 1587-1590. (10.1126/science.1237572) 23686338 PMC3713314

[134] Spillane KM, Tolar P. 2017 B cell antigen extraction is regulated by physical properties of antigen-presenting cells. J. Cell Biol. **216**, 217-230. (10.1083/jcb.201607064) 27923880 PMC5223605

[135] Bongrand P. 1999 Ligand-receptor interactions. Reports Prog. Phys. **62**, 921-968. (10.1088/0034-4885/62/6/202)

[136] Pierres A, Benoliel AM, Bongrand P. 1995 Measuring the lifetime of bonds made between surface-linked molecules. J. Biol. Chem. **270**, 26 586-26 592. (10.1074/jbc.270.44.26586) 7592881

[137] Pierres A, Prakasam A, Touchard D, Benoliel AM, Bongrand P, Leckband D. 2007 Dissecting subsecond cadherin bound states reveals an efficient way for cells to achieve ultrafast probing of their environment. FEBS Lett. **581**, 1841-1846. (10.1016/j.febslet.2007.03.077) 17434495 PMC1995029

[138] Shamri R et al. 2005 Lymphocyte arrest requires instantaneous induction of an extended LFA-1 conformation mediated by endothelium-bound chemokines. Nature Immunol. **6**, 497-506. (10.1038/ni1194) 15834409

[139] Mitrossilis D, Fouchard J, Pereira D, Postic F, Richert A, Saint-Jean M, Asnacios A. 2010 Real-time single-cell response to stiffness. Proc. Natl Acad. Sci. USA **107**, 16 518-16 523. (10.1073/pnas.1007940107) 20823257 PMC2944728

[140] Berridge MJ. 1997 The AM and FM of calcium signalling. Nature **386****,** 759-760. (10.1038/386759a0) 9126727

[141] Dolmetsch RE, Lewis RS, Goodnow CC, Healy JI. 1997 Differential activation of transcription factors induced by Ca^2+^ response amplitude and duration. Nature **386**, 855-858. (10.1038/386855a0) 9126747

[142] Dustin MJ, Bromley SK, Kan Z, Peterson DA, Unanue ER. 1997 Antigen receptor engagement delivers a stop signal to migrating T lymphocytes. Proc. Natl Acad. Sci. USA **94**, 3909-3913. (10.1073/pnas.94.8.3909) 9108078 PMC20541

[143] Forgacs G, Yook SH, Janmey PA, Jeong H, Burd CG. 2004 Role of the cytoskeleton in signaling networks. J. Cell Sci. **117**, 2769-2775. (10.1242/jcs.01122) 15150320

[144] Nature. 2000 Can biological phenomena be understood by humans? Nature **403**, 345. (10.1038/35000353) 10667755

[145] Service RF. 2020 The game has changed: AI triumphs at protein folding. Science **370**, 1144-1145. (10.1126/science.370.6521.1144) 33273077

[146] Mykuliak VV, Sikora M, Booth JJ, Cieplak M, Shalashilin DV, Hytönen VP. 2020 Mechanical unfolding of proteins—a comparative nonequilibrium molecular dynamics study. Biophys. J. **119**, 939-949. (10.1016/j.bpj.2020.07.030) 32822586 PMC7474207

[147] Loew LM, Schaff JC. 2001 The virtual cell: a software environment for computational cell biology. Trends Biotechnol. **19**, 401-406. (10.1016/S0167-7799(01)01740-1) 11587765

[148] Netz RR, Eaton WA. 2021 Estimating computational limits on theoretical descriptions of biological cells. Proc. Natl Acad. Sci. USA **118**, e2022753118. (10.1073/pnas.2022753118) 33495364 PMC8017709

[149] Fleetwood O, Kasimova MA, Westerlund AM, Delemotte L. 2020 Molecular insights from conformational ensembles via machine learning. Biophys. J. **118**, 765-780. (10.1016/j.bpj.2019.12.016) 31952811 PMC7002924

[150] Creighton TE. 1992. Proteins, 2nd edn. New York, NY: Freeman.

[151] Williams AF. 1991 Out of equilibrium. Nature **352**, 373-374. (10.1515/9783486783568-021) 1714038

[152] von Andrian UH, Chambers JD, McEvoy LM, Bargatze RF, Arfors KE, Butcher EC. 1991 Two-step model of leukocyte-endothelial cell interaction in inflammation: distinct roles for LECAM-1 and the leukocyte *β*2 integrins in vivo. Proc. Natl Acad. Sci. USA **88**, 7538-7542. (10.1073/pnas.88.17.7538) 1715568 PMC52336

[153] Lawrence MB, Springer TA. 1991 Leukocytes roll on a selectin at physiologic flow rates: distinction from and prerequisite for adhesion through integrins. Cell **65**, 859-873. (10.1016/0092-8674(91)90393-D) 1710173

[154] Kaplanski G, Farnarier C, Tissot O, Pierres A, Benoliel A-M, Alessi M-C, Kaplanski C, Bongrand P. 1993 Granulocyte-endothelium initial adhesion: analysis of transient binding events mediated by E-selectin in a laminar shear flow. Biophys. J. **64**, 1922-1933. (10.1016/S0006-3495(93)81563-7) 7690258 PMC1262526

[155] Alon R, Hammer DA, Springer TA. 1995 Lifetime of the P-selectin-carbohydrate bond and its response to tensile force in hydrodynamic flow. Nature **374**, 539-542. (10.1038/374539a0) 7535385

[156] Vitte J, Benoliel AM, Pierres A, Bongrand P. 2004 Is there a predictable relationship between surface physical-chemical properties and cell behaviour at the interface? Eur. Cells Mater. **7**, 52-63. (10.22203/eCM.v007a06) 15389394

[157] Pierres A, Touchard D, Benoliel AM, Bongrand P. 2002 Dissecting streptavidin-biotin interaction with a laminar flow chamber. Biophys. J. **82**, 3214-3223. (10.1016/S0006-3495(02)75664-6) 12023246 PMC1302111

[158] Bell GI. 1978 Models for the specific adhesion of cells to cells. Science **200**, 618-627. (10.1126/science.347575) 347575

[159] Chen S, Springer TA. 2001 Selectin receptor-ligand bonds: formation limited by shear rate and dissociation governed by the Bell Model. Proc. Natl Acad. Sci. USA **98**, 950-955. (10.1073/pnas.98.3.950) 11158576 PMC14690

[160] Foote J, Milstein C. 1994 Conformational isomerism and the diversity of antibodies. Proc. Natl Acad. Sci. USA **91**, 10 370-10 374. (10.1073/pnas.91.22.10370) PMC45021 7937957

[161] Zwanzig R. 1988 Diffusion in a rough potential. Proc. Natl Acad. Sci. USA **85**, 2029-2030. (10.1073/pnas.85.7.2029) 3353365 PMC279921

[162] Robert P, Limozin L, Pierres A, Bongrand P. 2009 Biomolecule association rates do not provide a complete description of bond formation. Biophys. J **96**, 4642-4650. (10.1016/j.bpj.2009.03.020) 19486686 PMC2711500

[163] Robert P, Nicolas A, Aranda-Espinoza S, Bongrand P, Limozin L 2011 Minimal encounter time and separation determine ligand-receptor binding in cell adhesion. Biophys. J. **100**, 2642-2651. (10.1016/j.bpj.2011.04.011) 21641309 PMC3117183

[164] Marshall BT, Sarangapani KK, Lou J, McEver RP, Zhu C. 2005 Force history dependence of receptor-ligand dissociation. Biophys. J. **88**, 1458-1466. (10.1529/biophysj.104.050567) 15556978 PMC1305147

[165] Pincet F, Husson J. 2005 The solution of the streptatividin-biotin paradox: the influence of history on the strength of single molecular bonds. Biophys. J. **89**, 4374-4381. (10.1529/biophysj.105.067769) 16169976 PMC1367001

[166] Lo Schiavo V, Robert P, Limozin L, Bongrand P. 2012 Quantitative modeling assesses the contribution of bond strengthening, rebinding and force sharing to the avidity of biomolecule interactions. PLoS ONE **7**, e44070. (10.1371/journal.pone.0044070) 23024747 PMC3443103

[167] Gonzalez C, Chames P, Kerfelec B, Baty D, Robert P, Limozin L. 2019 Nanobody-CD16 catch bond reveals NK cell mechanosensitivity. Biophys. J. **116****,** 1516-1526. (10.1016/j.bpj.2019.03.012) 30979550 PMC6486492

[168] Finger EB, Puri KD, Alon R, Lawrence MB, von Andrian UH, Springer TA. 1996 Adhesion through L-selectin requires a threshold hydrodynamic shear. Nature **379**, 266-269. (10.1038/379266a0) 8538793

[169] Thomas WE, Trintchina E, Forero M, Vogel V, Sokurenko EV. 2002 Bacterial adhesion to target cells enhanced by shear force. Cell **109**, 913-923. (10.1016/S0092-8674(02)00796-1) 12110187

[170] Marshall BT, Long M, Piper JW, Yago T, McEver RP, Zhu C. 2003 Direct observation of catch bonds involving cell-adhesion molecules. Nature **423**, 190-193. (10.1038/nature01605) 12736689

[171] Dembo M, Torney DC, Saxman K, Hammer D. 1988 The reaction-limited kinetics of membrane-to-surface adhesion and detachment. Proc. R. Soc. Lond. B **234**, 55-83. (10.1098/rspb.1988.0038) 2901109

[172] Lee C-Y et al. 2013 Actin depolymerization under force is governed by lysine 113:glutamic acid 195-mediated catch-slip bonds. Proc. Natl Acad. Sci. USA **110**, 5022-5027. (10.1073/pnas.1218407110) 23460697 PMC3612643

[173] Buckley CD, Tan J, Anderson KL, Hanein D, Volkmann N, Weis WI, Nelson WJ, Dunn AR. 2014 The minimal cadherin-catenin complex binds to actin filaments under force. Science **346****,** 1254211. (10.1126/science.1254211) 25359979 PMC4364042

[174] Luca VC et al. 2017 Notch-Jagged complex structure implicates a catch bond in tuning ligand sensitivity. Science **355**, 1320-1324. (10.1126/science.aaf9739) 28254785 PMC5459593

[175] Hong J et al. 2018. A TCR mechanotransduction signaling loop induces negative selection in the thymus. Nat. Immunol. **19**, 1379-1390. (10.1038/s41590-018-0259-z) 30420628 PMC6452639

[176] Huang S. 2009 Reprogramming cell fates: reconciling rarity with robustness. BioEssays **31**, 546-560. (10.1002/bies.200800189) 19319911

[177] Venkatachalapathy H, Azarin S, Sarkar CA. 2021 Trajectory-based energy landscapes of gene regulatory networks. Biophys. J. **120**, 687-698. (10.1016/j.bpj.2020.11.2279) 33453275 PMC7896034

[178] Stone L, Simberloff D, Artzy-Randrup Y. 2019 Network motifs and their origins. PLoS Comp. Biol. **15,** e1006749. (10.1371/journal.pcbi.1006749) PMC6459481 30973867

[179] Zaidel-Bar R, Itzkovitz S, Ma'ayan A, Iyengar R, Geiger B. 2007 Functional atlas of the integrin adhesome. Nat. Cell Biol. **9**, 858-867. (10.1038/ncb0807-858) 17671451 PMC2735470

[180] Thomas JD, Lee T, Suh NP. 2004 A function-based framework for understanding biological systems. Annu. Rev. Biophys. Biomol. Struct. **33**, 75-93. (10.1146/annurev.biophys.33.110502.132654) 15139805

[181] Naldi A, Carneiro J, Chaouiya C, Thieffry D. 2010 Diversity and plasticity of Th cell types predicted from regulatory network modelling. PLoS Comput. Biol **6**, e1000912. (10.1371/journal.pcbi.1000912) 20824124 PMC2932677

[182] Cho M-H, Wrabl JO, Taylor J, Hilser VJ. 2021 Hidden dynamic signatures drive substrate selectivity in the disordered phosphoproteome. Proc. Natl Acad. Sci. USA **117**, 23 606-23 616. (10.1073/pnas.1921473117) PMC7519349 32900925

[183] Rip J, de Bruijn MJW, Kaptein A, Hendriks RW, Corneth OBJ. 2020 Phosphoflow protocol for signaling studies in human and murine B cell subpopulations. J. Immunol. **204**, 2852-2863. (10.4049/jimmunol.1901117) 32253241

